# Recentralizing healthcare through evidence-based guidelines – striving for national equity in Sweden

**DOI:** 10.1186/s12913-014-0509-1

**Published:** 2014-11-05

**Authors:** Mio Fredriksson, Paula Blomqvist, Ulrika Winblad

**Affiliations:** Department of Public Health and Caring Sciences, Health Services Research, Uppsala University, Box 564, 751 22 Uppsala, Sweden; Department of Government, Uppsala University, Box 514, 751 20 Uppsala, Sweden

**Keywords:** Evidence-based guidelines, National guidelines, Governance, Healthcare regulation, Recentralization, Sweden, Equity, Cost-effectiveness, Prioritizations

## Abstract

**Background:**

The Swedish government has increasingly begun to rely on so called informative governance when regulating healthcare. The question this article sets out to answer is: considered to be “the backbone” of the Swedish state’s strategy for informative governance in healthcare, what kind of regulatory arrangement is the evidence-based National Guidelines? Together with national medical registries and an extensive system of quality and efficiency indicators, the National Guidelines constitutes Sweden’s quality management system.

**Methods:**

A framework for evaluating and comparing regulatory arrangements was used. It asks for instance: what is the purpose of the regulation and are regulation methods oriented towards deterrence or compliance?

**Results:**

The Swedish National Guidelines is a regulatory arrangement intended to govern the prioritizations of all decision makers – politicians and administrators in the self-governing county councils as well as healthcare professionals – through a compliance model backed up by top-down benchmarking and built-in mechanisms for monitoring. It is thus an instrument for the central state to steer local political authorities. The purpose is to achieve equitable and cost-effective healthcare.

**Conclusions:**

This article suggests that the use of evidence-based guidelines in Swedish healthcare should be seen in the light of Sweden’s constitutional setting, with several autonomous levels of political authority negotiating the scope for their decision-making power. As decision-making capacity is relocated to the central government – from the democratically elected county councils responsible for financing and provision of healthcare – the Swedish National Guidelines is part of an ongoing process of healthcare recentralization in Sweden, reducing the scope for local decision-making. This represents a new aspect of evidence-based medicine (EBM) and clinical practice guidelines (CPGs).

**Electronic supplementary material:**

The online version of this article (doi:10.1186/s12913-014-0509-1) contains supplementary material, which is available to authorized users.

## Background

Evidence-based medicine (EBM) has become an essential part of medical decision-making. Clinical practice guidelines (CPGs), and similar systematic approaches to specifying “best practices”, are at present the primary method for implementing EBM in healthcare practice [1]. CPGs are generally defined in terms of quality and efficiency improvement and thought to help practitioners and patients make informed decisions about appropriate healthcare for specific clinical circumstances [2]. EBM and CPGs are also part of health policy [3-5], and inversely, there is a progress towards evidence-based policymaking. In this article we take a closer look at governmental use of CPGs, more specifically how Swedish National Guidelines is used as one of the national state’s instruments for governing healthcare [6]. We show that the regulatory design of the Swedish National Guidelines allows for central control of local political and clinical decision-making, thus limiting the scope for decentralized healthcare.

Although the development of CPGs started out as an intra-professional endeavor [7], and in many respects still is, other actors are today involved in the production of CPGs. The best known European example is the National Institute for Health and Care Excellence (NICE), an “independent organisation responsible for developing national guidance, standards and information on providing high-quality health and social care, and preventing and treating ill health” in the UK [8]. In Sweden, a government agency – the National Board of Health and Welfare (NBHW) – develops so called *National guidelines for healthcare*; evidence-based decision supports that consist of recommendations for prevention, diagnosis and treatment of diseases that affect large numbers of patients and are costly to society. The National guidelines are considered “the backbone” of the state’s strategy for *informative governance* in healthcare, which also comprises e.g. the National Quality Registries, the Regional Comparisons of Quality and Efficiency in Swedish Health Care, the web-based health library for health professionals, and systematic knowledge reviews produced by government agencies. Informative governance is a system that seeks to increase the extent of evidence-based practice [9] through the developing, dissemination and implementation of best available knowledge to achieve the best possible benefit to users and patients [10]. It differs from EBM as it includes activities to create and maintain structures and processes that lead to the use of best available knowledge when making clinical and administrative decisions [11]. Structures (such as collaborative forums for the government agencies involved in informative governance) and processes created to develop and implement guidelines are central to the Swedish state’s informative governance strategy, but it also includes e.g. support for long-term knowledge development in the health service and at the Universities, and systems for the monitoring and public presentation of results from the health service. The Swedish government is increasingly relying on informative governance in governing and regulating healthcare, alongside legislation, supervision, economic incentives, agreements, monitoring and evaluation [12]. Several government agencies have recently intensified its informative governance approach [10]. The approach is akin to clinical governance, first used by the UK department of health [13].

Healthcare being a complex field of services, governments today control producers and professionals through a multitude of regulatory arrangements with varying force or capacity. How evidence-based guidelines fit into this variety of regulatory arrangements is a question that deserves more attention, not least because governance based on EBM may have a direct impact on patients’ health and quality of life. Informative governance in Sweden is not intended to replace other forms of governance, which implies governmental control based on EBM has some unique governing functions or features. This leads to questions about who is to be governed, in what way, and why; questions that fundamentally concern the design of the regulatory arrangement.

Thus, in the present article we use a framework for evaluating and comparing regulatory arrangements to make an analysis of the Swedish National guidelines [14]. In essence, the question this article sets out to answer is: considered to be “the backbone” of the state’s strategy for informative governance, what kind of regulatory arrangement is the Swedish National Guidelines? For instance, what is the purpose of the regulation, what organizations and activities are subject to regulation, are regulation methods oriented towards deterrence or compliance, and what methods are used for monitoring compliance? These questions are asked within the context of a decentralized healthcare system where the responsibilities for healthcare are divided between three different governing levels: the central state, county councils/regions (both of which are referred to as county councils in the article) and municipalities. The state is responsible for overall health care policies whereas the 21 county councils are responsible for the funding and delivery of healthcare and the 290 municipalities are responsible for care for the elderly and the disabled, as well as long-term psychiatric care.

## Method

In this article we use a framework for evaluating and comparing regulatory arrangements elaborated by Walshe and Shortell [14]. It is based on seven main areas for evaluation, see Table 1. It can be used both for conceptual evaluations or to develop more quantitative measures.

**Table 1:** **Framework for evaluating regulation**

**Characteristic**	**Questions**	**Concerns**
Regulating organization	What kind of organization takes on the role of regulator?	Constitution, formal mandate, reporting or accountability arrangements etc.
Regulatory goal/objectives	What is the purpose of regulation and how explicitly it is stated?	The range of problems or needs addressed
Scope of regulation	What organizations and activities are subject to regulation?	Forms or types of organization (horizontal scope)
		Which of their functions or activities (vertical scope)
Regulatory model	Are regulation methods oriented towards deterrence or compliance?	Deterrence: distant, formal adversarial relations, formal sanctions and penalties
		Compliance: close, friendly relationships, support, educational activities etc.
Regulatory direction	How are regulatory requirements communicated?	Written standards, guidance on implementation etc.
Regulatory detection	What methods are used for monitoring compliance?	Surveys, inspections, data sources, follow-ups etc.
Regulatory enforcement	What methods are used for enforcement?	Disclosure of findings, incentives, sanctions, financial penalties etc.

Source: Walshe K. Regulating healthcare - A prescription for improvement? Maidenhead: Open University Press; 2003. For clarification purposes some adjustments of Walshe and Shortells’ original model have been made, but no changes of model content.

By making a qualitative document analysis, we investigate how the idea and intent of the Swedish National Guidelines is expressed in public documents and information material emanating from the National Board of Health and Welfare (NBHW), the Government and the state administration. Qualitative document analysis is a systematic procedure used for making empirical observations based on written records [15]. In the public documents we have searched answers to the seven questions in Walshe and Shortells’ model. The final analysis is based on 25 documents, indicated by superscript figures in the text, and listed in Additional file 1. Most important are two different documents from the NBHW describing development and intent ^1,7^ and the information material presented at the website of NBHW (www.socialstyrelsen.se).

As mentioned, several Swedish government agencies operating within the field of healthcare are involved in informative governance. The National Board of Health and Welfare is, however, solely responsible for the development of National guidelines, see Table 2. Thus, the article does not address the activities of agencies such as the Dental and Pharmaceutical Benefits Agency (the TLV) that determines whether a pharmaceutical product or dental care procedure is subsidized by the state, or the Swedish Council on Health Technology Assessment (SBU). SBU’s assessments of health care interventions are however important sources of information when the NBHW develops National Guidelines.

**Table 2:** **Swedish National Guidelines autumn 2014**

Published guidelines	• National Guidelines for the Treatment of Breast, Prostate and Colorectal Cancers (1,2,5,6)
	• National Guidelines for Antipsychotic Drug Therapy for Schizophrenia or Schizophrenia-type Conditions (1,5,6)
	• National Guidelines for Palliative care (5,6)
	• National Guidelines for Musculoskeletal Diseases (1,6)
	• National Guidelines for Methods of Preventing Disease (1,3,4,5,6)
	• National Guidelines for Adult Dental Care (1,3,6)
	• National Guidelines for Lung Cancer Care and Treatment (1,2,3,6)
	• National Guidelines for Psychosocial Interventions for Schizophrenia or Schizophrenia-type Conditions (1,3,4,6)
	• National Guidelines for Care in cases of Dementia (1,3,4,6)
	• National Guidelines for Care in cases of Depression and Anxiety Disorders (1,3,4,6)
	• National Guidelines for Stroke Care (1,2,3,6)
Preliminary guidelines	• National guidelines for Diabetes Care (final version finished spring 2015)
	• National guidelines for Substance Abuse Treatment (final version finished spring 2015)
Updates and new guidelines	• National guidelines for Asthma and COPD (preliminary version finished autumn 2014)
	• National guidelines for Cardiac Care (preliminary version finished spring 2015)
	• National guidelines for Multiple sclerosis and Parkinson’s disease (preliminary version finished spring 2016)

Table explanation. The numbers (1–6) refer to implementation facilitators accessible at the website of the National Board of Health and Welfare. Implementation facilitators: 1. Indicators, 2. Target levels, 3. Patient version of guideline, 4. Implementation support, e.g. web-based tutorial, 5. Interactive information material, 6. Additional resources, e.g. professional views, survey material. Source: http://www.socialstyrelsen.se (27-09-2014).

### Ethics statement

No ethics approval was needed according to the Declaration of Helsinki as the study does not involve human subjects, or identifiable human material and data.

## Results

### Regulating organization

The NBHW – a government agency under the jurisdiction of the Ministry of Health and Social Affairs – is today commissioned by the government to *develop* and *monitor* the Swedish National Guidelines as well as to *support implementation*^1^. New guideline areas are sometimes a direct mandate from the government. The NBHW’s governance mandate is different from that of HTA organizations in Sweden, for instance the Swedish Council on Health Technology Assessment (SBU). SBU is involved in *knowledge mediating processes* (supporting the recipient’s own knowledge formation); while the NBHW is involved in *strategic intervention processes* (assessing knowledge aiming to influence the recipient’s decisions)^2^.

Government agencies in Sweden are considered important actors in the process of transforming political decisions into actual policy [16]. Key activities of the NBHW are to manage information with relevance for healthcare and to develop standards and issue regulation. Until June 2013 a key activity was also to supervise the health and medical services and staff^3^ and the NBHW then had the mandate to impose financial penalties or prohibit providers’ activities^4^. Supervision is now carried out by a separate agency, the Health and Social Care Inspectorate. The government influences the general direction of the activities of the NBHW through yearly instructions on how the agency may use the resources allocated to them by the parliament and what goals the agency is to achieve. The NBHW make Annual Reports to the Government. In the Annual Report from 2010, the National guidelines’ important function in the welfare system is pointed out. At the first page the Director General stated that “Equity in healthcare is a momentous issue for the Swedish welfare state and one of the issues the NBHW has explicitly pursued during the year /…/ the National guidelines help to make healthcare more equitable across the country”^5^.

The development of National guidelines – which we will not describe in detail here – consists of six steps; selection of guideline area, identification and scientific review of so called *condition-intervention pairs,* ranking of condition-intervention pairs, preliminary version of guidelines, final version of guidelines, and lastly, measuring and monitoring^1^. The development process is led by professional project managers from the NBHW. It involves multi-professional expertise appointed by the NBHW, sometimes in collaboration with specialist associations, and represents the whole chain of care^1,6^. When developing National guidelines on heart disease, the process involved physicians within the relevant specialties but also general practitioners, nurses, physiotherapists, ethicists and health economists, see [17]. Thus, multi-professional expertise is involved, but the development process is set by the NBHW that is responsible for the guideline content and recommendations, which must be authorized by the Director General at the NBHW^7^. The NBHW claims the legitimacy of National guidelines is dependent on that the NBHW is perceived as a legitimate regulator and that the engaged experts have a good reputation^1^.

If professional associations produce care plans or guidelines, the NBHW claims that it is important that those guidelines complement the Swedish National Guidelines rather than compete with them^8^. When a National Guideline is published, it becomes the “gold standard” within that disease group in Sweden. Thus, the NBHW has a superior position as guideline producer in Sweden.

### Regulatory goal/objectives

In 1996, the NBHW was first assigned to develop National guidelines for Swedish healthcare, at that time called National healthcare programs. Variations in medical praxis were considered unsatisfactory both from a resource point of view, and in light of the equity-objective of “good healthcare on equal terms” stated in the Swedish Health and Medical Services Act (1982:763). National healthcare programs were considered a way to strengthen patients’ possibilities of receiving equivalent and evidence-based healthcare throughout Sweden^9^. Fifteen years later the goals are largely the same, except from the new emphasis on *prioritizations* (see Scope of regulation).

In the studied documents, it is possible to find several formulations about objectives. At the website, the NBHW states that the National guidelines will “contribute to strengthening the possibilities for the people to receive equal and good healthcare”^10,12^. Such a formulation corresponds directly to the national Health and Medical Services Act (1982:763) establishing that “the goal of all healthcare services is good health and healthcare on equal terms for the entire population”. According to the NBHW, evidence is a prerequisite for achieving good healthcare, and the agency establishes that informative governance will lead to more equivalent healthcare provision regardless of where in Sweden one lives and seeks care^8^. Thus, the National guidelines are intended to “even out geographical differences in the quality of care”^11^.

*Effective use of healthcare resources* is another clearly pronounced objective of the National guidelines. The NBHW establishes that Swedish National Guidelines are intended to support the production of “cost-effective healthcare that puts patient benefit first”^1^ also considering “the needs of the population so that resources achieve the maximum possible benefit”^13^. It is thus a combination of the point of view of the patient and the community. In Swedish regulation, the objective of cost-effectiveness is most clearly formulated in the ethical platform established by the Parliament in 1997 (see Scope of regulation), which stipulates that, when choosing between treatments or interventions, there should be a reasonable relationship between costs and effectiveness in terms of health and quality of life. Cost-effectiveness is thus an ethical principle and healthcare professionals and principals are obliged to use resources to achieve the best effect – anything else is considered unethical. However, saving money is not an explicit goal of the Swedish National Guidelines compared to, for instance, NICE clinical guidelines that provides cost saving guidance [18].

### Scope of regulation

#### Making priorities – the vertical scope

According to the NBHW, the Swedish National Guidelines are intended as a support for prioritizations and decision-making on how to allocate resources within healthcare according to population need^1^. The Swedish National Guidelines is the NBHW’s primary way of working with prioritizations, and of implementing the national model for prioritizations established by the parliament in 1997^14,15,16^.

Today, each National guideline contains a number of recommendations with an established degree of priority (from 1–10), crucial for decision-making and prioritization. Simply put, an intervention with priority 1 is always a higher priority than an intervention with priority 2 or 3, and should accordingly be allocated more resources. This applies to political decision-makers as well as to health professionals, as we show in the next section. For example, in the National Guidelines for Stroke Care (2009), one of the recommendations (priority 1) is that the health service shall promote the immediate diagnosis and treatment of patients suffering from a suspected TIA (transient ischemic attack) at emergency wards by physicians specializing in strokes^17^. The ranking of recommendations draws on the identification and scientific review of so-called condition-intervention pairs, (e.g. 164 pairs in the National Guidelines for Stroke Care mentioned above). The degree of priority is then determined by combining the severity of the condition, the effects of the intervention and cost-effectiveness of the intervention (often expressed as QALYs). The scientific strength of the evidence for effects and cost-effectiveness is also included in the ranking, as are ethical concerns. The leading idea behind the ranking is that highly ranked interventions shall receive a large part of the resources, whereas low ranking interventions shall receive a small part. As an example, highly ranked condition-intervention pairs are often of great benefit to patients and cost-effective for society. Condition-intervention pairs with an inadequate scientific base are low ranking^1^.

#### A decision-support for all decision-makers – the horizontal scope

Politicians, leading administrators, healthcare managers and healthcare professionals are the target groups that are intended to use the Swedish National Guidelines^18^. The opinion of the NBHW is that decision-makers must take a standpoint on how to allocate resources within and between different areas of the healthcare system^10^. More explicitly, the Swedish National Guidelines can be used for 1) decisions on resource allocation within and between different groups and treatment areas, 2) decisions on management and organizational planning, 3) decisions on local and regional healthcare programs and 4) individual decisions made by physicians meeting individual patients^18^. Hence, both horizontal prioritizations (political priorities between disease groups) and vertical prioritizations (medical priorities within a disease group) are the scope. The intended use for political or administrative decisions is, however, more emphasized in the studied documents than is clinical use. It is specifically mentioned in the final guideline documents that the guideline recommendations are intended to support decisions at the group level ^e.g., 17^. In comparison, the NICE clinical guidelines aims “to help patients to make informed decisions” and to “improve communication between patient and health professional” [19].

Thus, the guideline recommendations are intended to serve as a basis for politicians and health directors when they are making decisions about resource allocation as well as organization and competence improvement^19^. In fact the NBHW encourages a development toward closer integration of the Swedish National Guidelines into budgetary processes in county councils^16^. Some recommendations are calculated to have structural consequences, such as regarding the need for new medical skills or new medical equipment. Such changes often involve major reallocation of resources and allocation of additional resources, which must be decided by local political government^19,20,7^. In the TIA-example mentioned earlier, the NBHW made the assessment that implementation will increase the pressure on emergency wards and increase the number of hospital beds needed, which initially will lead to increased costs. The NBHW also calculated the need for supplementary training programs for physicians. The guideline recommendations may thus free up resources by limiting the practice of certain treatments, but also require *additional* resources when introducing new interventions^1^. For example, in the National Guidelines for Stroke Care, the NBHW presents a summary including ten recommended interventions. Four interventions imply cost savings and six interventions are associated with cost increases, at least initially. Six interventions involve no organizational impact, whereas four interventions require organizational change^17^.

### Regulatory model and regulatory direction

The regulatory model is consistent with the so called compliance-model, which means the NBHW and the county councils share important goals. For instance, the regulator and regulated organizations share the objective of “healthcare on equal terms” for the whole Swedish population (Health and Medical Services Act 1982:763) and provision of healthcare in accordance with “science and proven experience” (Patient Safety Act 2010:659).

The compliance model is visible throughout the entire guideline process involving health professionals in the development stages, as well as the county councils in the implementation. The preliminary guideline versions are open for dialogue with decision makers, professions and patient organizations and discussed at conferences, seminars and hearings. The preliminary versions allow the county councils to make their own organizational and financial analyses of the guideline recommendations. The NBHW take these analyses into consideration when establishing the final guideline version^1,6^.

Mainly it is the county councils’ responsibility to implement the National guidelines, with support from the NBHW. The structure for implementation differs between the county councils, but implementation is a prioritized issue. The county councils’ willingness to implement the guidelines is reflected in their expressed need for an effective standard process for implementation^2^. The regulatory requirements are communicated openly by publishing all documents related to the final guideline versions^21^.

### Regulatory detection and regulatory enforcement

Guideline-specific sets of quality indicators for the most important recommendations are crucial for monitoring and follow-ups. The NBHW consider these quality indicators a necessary condition for efficient informative governance^22^. Parallel to the guideline development process an expert group develops quality indicators that are central to decision-makers. In 2009 it was pointed out that, as far as possible, guideline follow-ups shall be linked to medical registry-data, e.g. the *National Quality Registries* (contains personalized information about problems/diagnosis, treatment, and results) and the Regional Comparisons of Quality and Efficiency in Swedish Health Care^8^.

Monitoring and follow-ups are part of the regulatory enforcement. The NBHW evaluates how National guidelines are used in the county councils and presents the results openly^23^, which in practice create strong incentives for implementing the National guidelines. For instance in 2011, the NBHW presented a National evaluation of Stroke care, presenting the results of each county council in comparison to the national average, at both county council and hospital level^25^. The use of national quality-indicators may be seen as top-down bench-marking as it is “imposed from above” [20], i.e. established by the NBHW rather than the county councils themselves. The National guidelines are not legally binding but “strongly directing”^24^. The NBHW emphasizes that all involved in resource allocation, management and organizational planning as well as decisions about the treatment of individual patients should have good knowledge of the content and impact of the recommendations within the National guidelines^18^. In practice, this is a strong incentive to follow the recommendations.

## Discussion

The findings from the previous analysis suggest that the idea of the Swedish National Guidelines is to govern all decision makers – politicians and administrators in the county councils as well as healthcare professionals – through a compliance model backed up by top-down benchmarking and built-in mechanisms for monitoring. The purpose is to achieve equitable and cost-effective healthcare in the whole country. One of the consequences, however, is a weakening of local self-government.

The regulatory scope is what gives the Swedish National Guidelines their capacity to influence decision making at all levels involved in provision of healthcare. The scope of regulation is both meso-level prioritizations (county council politicians and administrators) and micro-level prioritizations (healthcare professionals). Thus, apart from being a channel for professional guidance, we find the Swedish National Guidelines to be a regulatory arrangement making it possible for the national state to govern local political authorities and to take on a clearer role in assuring care quality [21].

The National Guidelines are developed and implemented in a healthcare system where responsibility for healthcare is divided between three political levels of government. All three levels of government are directly elected every four years and self-governing with taxation rights. From the early 1980s, the Swedish state has gradually shifted powers and authority downwards to the county councils and municipalities, building a strongly decentralized healthcare system [22]. The combination of locally elected political bodies and the possibility to raise local taxes distinguishes Sweden from the more centralized tax-based NHS [23]. As pointed out by, for example, Saltman [24], decentralization in European health systems has led to increased disparities in services provided and heightened equity problems. Sweden is no exception. Regional variations in healthcare utilization, quality and outcomes have repeatedly been found in national surveys, e.g. [16,25,26]. The 2013 Regional Comparisons of Quality and Efficiency in Swedish Health Care concludes that – although the majority of the indicators show improvement over time – there are variations between county councils, some of which are significant; a fact that point to there being scope for improvement of most of the 162 indicators. The regional variations seen in Sweden are in part associated with differences in the organization of healthcare production across county councils [27]. On the one hand, regional differences could be seen as a relevant adaptation to local conditions [28], for example demographic structure and morbidity patterns. On the other hand, regional differences could be seen as a possible threat to national equity [29]. During the 2000s, the opinion that differences between county councils might threaten the equity objective has come to dominate the political debate in Sweden, reflected in the National guidelines’ purpose of improving equity in access to effective methods for treatment.

Vrangbæk [28] proposes that three functional areas in healthcare can potentially range from being centralized to decentralized; *arranging/planning* (i.e. the regulatory function), *financing* and the *delivery* of healthcare services. According to the National Health and Medical Services Act (1982:763) as well as the Local Government Act (1991:900), county councils have the mandate to allocate resources and arrange services in order to “provide good healthcare to their inhabitants”. Establishing recommended interventions, the Swedish National Guidelines thus reduce the county councils’ discretion to define what good healthcare is and how to deliver it according to local conditions and preferences, which involves the allocation of resources. The Swedish National Guidelines may thus be seen as an attempt to intervene in the decentralized responsibilities for healthcare.

The National Guidelines affect the power and responsibility – linked to the range of decisions that can be made locally– in all functional areas. As the Swedish National Guidelines are part of an emerging centralized regulatory framework defining e.g. quality and service levels, the scope for local planning is curtailed. Furthermore, in practice, the Swedish National Guidelines also curtail the scope for making autonomous service-related decisions, by defining what technologies and processes to use and how to organize delivery, e.g. how to compose the workforce see [28]. In practice, the Swedish National Guidelines also affect resource allocation through its recommendations, although the county councils are still responsible for funding through local taxation. Thus, we find the Swedish National Guidelines a power-transfer arrangement altering the balance between national, regional and local levels of government. In a broader perspective the Swedish National Guidelines are part of a recentralization process already seen in other Nordic countries [23] and in other welfare sectors [30]. We thus suggest that the use of evidence-based guidelines is a way to retake power decentralized to the county councils the past 30 years, cf. [28]. Not to forget, the National guidelines also influence micro-level prioritizations made by health professionals. It has been suggested that guidelines threaten professional autonomy being an attempt to preset the actions of health professionals [31]. Peckham et al., for instance,[32] see changes in professional autonomy as directly linked to degree of decentralization in health services, implying that recentralization through National guidelines decreases professional autonomy.

The NBHW governs through a regulatory model oriented towards compliance, i.e. cooperation and goal-sharing between the regulator, the county councils, health professionals and experts. As the regulatory arrangement builds on compliance and cooperation, it is reasonable to assume that the regulatory aspects may not be as visible to those governed. The compliance model requires that there is at least some degree of willingness to implement the regulation, which in this specific case, can be linked to the guidelines’ perceived legitimacy. The cooperation strategy and the involvement of representatives from the health service largely contribute to the legitimacy (reducing affective barriers, see below). For instance, it has been reported that orthopedists consider it very important to comply with National guidelines in contrast to “political reforms” such as the Waiting-time guarantee [33]. Although the successful implementation of guidelines is considered to improve quality of care, it has been suggested that guidelines have had limited effect on changing physician behavior [34-36]. Barriers to guideline adherence have been related to physician *knowledge* (cognitive barriers such as lack of awareness and familiarity), *attitude* (affective barriers such as lack of agreement with guidelines in general or specific guidelines, and lack of outcome expectancy), and *behavior* (ability barriers such as lack of time and resources and organizational constraints) [35]. There are examples of interventions that have reduced barriers, e.g. [37], but several studies indicate the need for improved adherence e.g. [38,39]. To enhance implementation and adherence, the county councils have expressed desire for a nationally consistent implementation process indicating they perceive National guidelines as legitimate instruments for organizing provision at local level.

The compliance model may be seen as a version of consensus governance through soft-law practices, which is relatively widespread in Swedish healthcare [30,40]. Soft-law refers to explicit but not legally binding rules that are enforced by a variety of mechanisms, although lacking formal sanctions. One of its advantages is that it can allow for regulation where hard regulation would be impossible [41], for instance in an area controlled by strong professions and autonomous local governments with responsibility for healthcare provision. Soft law is often enforced through benchmarking, follow-ups and evaluations. Similarly, the analysis shows that the compliance model is complemented by an increasing reliance on measurements, monitoring and follow-ups based on quality indicators specifically tailored for each guideline. These results are made public, creating pressure to comply. Walshe and Shortell [14] point out that regulators often use disclosure or publication of results as an enforcement strategy. As such, National Guidelines are part of a multi-level approach of clinical governance interlinking several data sources for national benchmarking and quality control. The most explicit regulatory objectives are equitable distribution of quality healthcare and cost-effectiveness in healthcare. Used for health policy purposes, CPGs are often associated with a fear of rationalization and cost cutting [3,31]. However, cost-effectiveness *per se* does not have anything to do with saving money. A more expensive intervention may be more cost-effective than a less expensive one. Thus, increased costs may be the best use of resources. Rationing implies conscious limitations of the possibilities to optimally meet needs for healthcare, for instance by *limiting supply* (e.g. some conditions are excluded from public financing or fewer treatment sessions are offered) or by *limiting quality* (e.g. less expensive treatments or medication are offered) [42]. As some of the National guideline recommendations entail a need for additional resources, it seems that it is not a regulatory arrangement created for rationalization.

The results presented in the article are based on the study of documents only, which is a potential limitation. The documents contribute with information that expresses the state’s and the government agencies’ official approach – which is not necessarily equivalent to the actual practice of developing and implementing the National Guidelines. Thus, the actual practice may differ from the intent. Undoubtedly interviews would nuance the NBHW’s role and its intentions with the National guidelines, and also illustrate how local authorities and health professionals perceive of the guidelines’ governing functions. Such interviews could also illustrate how the development and implementation of the guidelines is carried out at various levels of the health care system. However, the aim of the study was to investigate what kind of regulatory arrangement the Swedish National Guidelines is (the idea and intent of the guidelines), not to investigate the implementation process or outcomes. Even so, quantitative data illustrating how the county councils perform on some of the recommendations presented in the guidelines, and whether differences between county councils have decreased since the guidelines were introduced, would point to the National guidelines’ actual regulatory impact - which is lacking in the current study. Using documents as the empirical source is, however, also a strength as a longer period of time can be accurately covered (in this case the development of the regulatory arrangement from 1997 and onwards), avoiding, for example, the recall bias associated with interviews. Another strength of the study is the use of an analytical model – Walshe and Shortells’ framework [14] for evaluating and comparing regulatory arrangements. Advantages of using an analytic model are that the analysis becomes more systematic and transparent, and that it is easier to compare the investigated case with other cases and to make theoretical connections and generalizations. Here, it enables comparisons with other types of regulation as well as with other types of clinical practice guidelines throughout the world.

## Conclusions

International comparisons have established that there is cross-country variation regarding the development, dissemination and implementation of CPGs [43]. The results presented here imply that Sweden, through its use of CPGs, is departing from a governance model which gives professional groups and local and regional authorities substantial autonomy in monitoring and improving their own standards of practice [21]. We suggest that the use of evidence-based guidelines in Swedish healthcare should be seen in light of the constitutional setting, with several autonomous levels of political authority that are constantly negotiating the space for their decision-making power. Thus, the Swedish case suggests that evidence-based guidelines can be used to regulate intra-state conditions, something that is normally not discussed in the literature on EBM and CPGs. Thus, the article makes its theoretical contributions at the intersection of EBM, CPGs and healthcare regulation. However, there is a lack of research on the effects of this specific regulatory arrangement in Sweden. Next step would be to study political and administrative use, for instance the impact in budget processes at local level.

## Additional file

Additional file 1:
**Document study material referred to in the final analysis in “Recentralizing healthcare through evidence-based guidelines – striving for equity in Sweden”.** The language of the original documents and information material is Swedish.

## Abbreviations

EBM: Evidence-based medicine
CPG(s): Clinical practice guidelines(s)
NBHW: The National Board of Health and Welfare, Sweden
NICE: The National Institute for Health and Clinical Excellence, UK
