# Supplementary material for: Recentralizing healthcare through evidence-based guidelines – striving for national equity in Sweden
Source: BMC Health Serv Res. 2014 Nov 5;14:509. doi: 10.1186/s12913-014-0509-1 (PMC4226849; doi:10.1186/s12913-014-0509-1)
Supplement: Additional file 1: — Document study material referred to in the final analysis in “Recentralizing healthcare through evidence-based guidelines – striving for equity in Sweden”. The language of the original documents and information material is Swedish. [file 12913_2014_509_MOESM1_ESM.docx]

| **Additional file 1.**  Document study material referred to in the final analysis in “Recentralizing healthcare through evidence-based guidelines – striving for equity in Sweden”.  The language of the original documents and information material is Swedish. | |
| --- | --- |
| ^1^ | *Socialstyrelsens plattform för Nationella riktlinjer. Hälso- och sjukvård, tandvård och socialtjänst*, Socialstyrelsen 2008. NBHW internal document. |
| _2_ | *Mot en effektivare kunskapsstyrning - Kartläggning och analys av nationellt och regionalt stöd för en evidensbaserad praktik i hälso- och sjukvården*, 2009 Socialstyrelsen. NBHW Report (2009-10-124). <http://www.socialstyrelsen.se/publikationer2009/2009-10-124> |
| ^3^ | Start /English /About us / About the National Board of Health and Welfare  <http://www.socialstyrelsen.se/english/aboutus> Available online 2014-05-09 |
| ^4^ | Start /Tillsyn /Om tillsyn /Så går tillsynen till, Available online 2011-11-24  <http://www.socialstyrelsen.se/tillsyn/omtillsyn/sagartillsynentill> |
| ^5^ | Årsredovisning 2010 – Socialstyrelsen, Available online 2011-11-24  <http://www.socialstyrelsen.se/Lists/Artikelkatalog/Attachments/18245/2011-2-11.pdf> |
| ^6^ | Start /Riktlinjer /Nationella riktlinjer /Om Nationella riktlinjer /Så tar vi fram riktlinjerna  [http://www.socialstyrelsen.se/riktlinjer/nationellariktlinjer/omnationellariktlinjer/satarviframriktlinjerna](http://www.socialstyrelsen.se/riktlinjer/nationellariktlinjer/omnationellariktlinjer/satarviframriktlinjerna%20Available%20online%202011-11-24) Available online 2014-05-09 |
| ^7^ | *Huvudprocessbeskrivning för Nationella riktlinjer för god vård*, Internt styrdokument/Processbeskrivning, 00-121/2009-60. NBHW document. |
| ^8^ | *Hälso- och sjukvårdsrapport 2009*, Socialstyrelsen. NBHW Report (2009-126-72).  <http://www.socialstyrelsen.se/Lists/Artikelkatalog/Attachments/8496/2009-126-72_200912672_rev2.pdf> |
| ^9^ | *Dagmaröverenskommelse 1997* *– överenskommelse mellan staten och Sveriges Kommuner och Landsting om vissa ersättningar till hälso- och sjukvården*. Archive material. |
| ^10^ | Start /Riktlinjer /Nationella riktlinjer /Om Nationella riktlinjer. Available online 2011-11-24 <http://www.socialstyrelsen.se/riktlinjer/nationellariktlinjer/omnationellariktlinjer> |
| ^11^ | Start /English /About us /Our activities. Available online 2014-05-09  <http://www.socialstyrelsen.se/english/aboutus/ouractivities> |
| ^12^ | Start /Pressrum /Nyhetsarkiv /”Fem helt nya nationella riktlinjer.  Available online 2011-11-24  <http://www.socialstyrelsen.se/pressrum/nyhetsarkiv/femheltnyanationellariktlinjer> |
| ^13^ | Start /National Guidelines /About the guidelines, Available online 2014-05-09  <http://www.socialstyrelsen.se/nationalguidelines/abouttheguidelines> |
| ^14^ | *Riktlinjer för prioriteringar inom hälso- och sjukvård*, Riksrevisionen 2004. RiR 2004:9. State report. <http://www.riksrevisionen.se/PageFiles/560/RiR_2004_9.pdf> |
| ^15^ | *Proposition 2002/03:1*, Budgetpropositionen för 2003. Government document.  <http://www.regeringen.se/content/1/c4/30/79/ef6dc1ba.pdf> |
| ^16^ | *Proposition 2004/05:1*, Budgetpropositionen för 2005. Government document.  <http://www.sweden.gov.se/content/1/c6/02/97/44/5780efa4.pdf> |
| ^17^ | *Nationella riktlinjer för strokesjukvård 2009 - Stöd för styrning och ledning*. Guideline document. <http://www.socialstyrelsen.se/nationellariktlinjerforstrokesjukvard> |
| ^18^ | *”Så kan riktlinjerna användas”* (2011-05-30). NBHW website information.  <http://www.socialstyrelsen.se/riktlinjer/nationellariktlinjer/omnationellariktlinjer/sakanriktlinjernaanvandas> |
| ^19^ | Metod för Socialstyrelsens arbete med nationella riktlinjer (Depression och ångestsyndrom), 2010. Guideline document appendix. <http://www.socialstyrelsen.se/publikationer2010/2010-3-4/Documents/Bilaga_5_Metodbilaga.pdf> |
| ^20^ | Metod för Socialstyrelsens arbete med riktlinjer (Schizofreni), 2011. Guideline document appendix.  <http://www.socialstyrelsen.se/publikationer2011/2011-1-3/Documents/metodbilaga.pdf> |
| ^21^ | Start /Riktlinjer /Nationella riktlinjer, Available online 2014-05-09  http://www.socialstyrelsen.se/riktlinjer/nationellariktlinjer |
| ^22^ | *Prioriteringar i hälso- och sjukvården. Socialstyrelsens analys och slutsatser utifrån rapporten "Vårdens alltför svåra val?",* Socialstyrelsen 2007. NBHW report (2007-103-4). <http://www.socialstyrelsen.se/Lists/Artikelkatalog/Attachments/8951/2007-103-4_20071034.pdf> |
| ^23^ | *Hälso- och sjukvårdsrapport 2009*, Socialstyrelsen. NBHW Report (2009-126-72).  <http://www.socialstyrelsen.se/Lists/Artikelkatalog/Attachments/8496/2009-126-72_200912672_rev2.pdf> |
| ^24^ | Start /National Guidelines /How we draw up the guidelines, Available online 2014-05-09 <http://www.socialstyrelsen.se/nationalguidelines/howwedrawuptheguidelines> |
| ^25^ | Start /Frågor och svar /Nationella riktlinjer för vård... Available online 2014-05-09 http://www.socialstyrelsen.se/fragorochsvar/nationellariktlinjerforvardvid#anchor_3 |
